# A Wireless Implant for Gastrointestinal Motility Disorders

**DOI:** 10.3390/mi9010017

**Published:** 2018-01-02

**Authors:** Yi-Kai Lo, Po-Min Wang, Genia Dubrovsky, Ming-Dao Wu, Michael Chan, James C. Y. Dunn, Wentai Liu

**Affiliations:** 1 Niche Biomedical, LLC, Los Angeles, CA 90095, USA; 2 Department of Bioengineering, University of California, Los Angeles, CA 90095, USA; pominwang@g.ucla.edu (P.-M.W.); bodombodom@ucla.edu (M.-D.W.); chanmi168@gmail.com (M.C.); jdunn2@stanford.edu (J.C.Y.D.); wentai@ucla.edu (W.L.); 3 Department of Surgery, University of California, Los Angeles, CA 90095, USA; GDubrovsky@mednet.ucla.edu; 4 Department of Surgery, Stanford University, Stanford, CA 94305, USA

**Keywords:** gastrointestinal stimulation, motility, implant, neuromodulation, bioelectronics medicine, electroceuticals, wireless power and data telemetry

## Abstract

Implantable functional electrical stimulation (IFES) has demonstrated its effectiveness as an alternative treatment option for diseases incurable pharmaceutically (e.g., retinal prosthesis, cochlear implant, spinal cord implant for pain relief). However, the development of IFES for gastrointestinal (GI) tract modulation is still limited due to the poorly understood GI neural network (gut–brain axis) and the fundamental difference among activating/monitoring smooth muscles, skeletal muscles and neurons. This inevitably imposes different design specifications for GI implants. This paper thus addresses the design requirements for an implant to treat GI dysmotility and presents a miniaturized wireless implant capable of modulating and recording GI motility. This implant incorporates a custom-made system-on-a-chip (SoC) and a heterogeneous system-in-a-package (SiP) for device miniaturization and integration. An in vivo experiment using both rodent and porcine models is further conducted to validate the effectiveness of the implant.

## 1. Introduction

Gastrointestinal neuromuscular disorder (GND) is a set of disorders characterized by the absence or poor function of the intestinal muscularis (IM), involving any segment of the gastrointestinal (GI) tract. GND may affect the enteric nervous system, smooth muscle cells, and/or the interstitial cells of Cajal (ICC), which are the pacemaker cells in the GI tract, thus resulting in functional GI diseases and dysmotility. Patients with GND may present with dysphagia, gastroesophageal reflux disease (GERD), nausea, functional dyspepsia, blockage of transit, and obstruction of the GI tract (e.g., gastroparesis), which accounts for 40% of GI tract illness that patients seek health care for in gastroenterology clinics [1]. The current limitation in the treatment of GND associated GI dysmotility is the lack of understanding of the pathophysiology involving the neurons, ICC, and smooth muscle cells combined with the paucity of effective medications that can improve GI motility. The clinical alternative for pharmaceutically intractable GI dysmotility is usually the total or subtotal resection of the affected GI segments [2].

Pioneer work done by Bilgutay et al. has investigated the possibilities of using electrical stimulation on GI organs to improve GI motility dysfunctions [3]. More recently, gastric electrical stimulation (GES) using an implantable neurostimulator (Enterra Therapy ITREL 3 model 7425G; Medtronic Inc., Minneapolis, MN, USA), received a humanitarian device exemption for the management of patients suffering from nausea. Other works adopting functional electrical stimulation have also been shown as a potential treatment, including lower esophageal sphincter stimulation for GERD [4], gastric stimulation for obesity [5], intestinal stimulation for post-operative ileus management [6], and colon stimulation to control the movement of solid content using a multichannel stimulator [7]. Nonetheless, the clinical effectiveness and applicability of implantable GI electrical stimulation is somehow limited by the capabilities of existing implantable GI stimulators, including (1) limited programmability: it has been shown that a wide range of parameters (e.g., 0.2–100 ms, 10–50 mA, and 3–1670 Hz) can be used to induce GI contraction [8], but no existing implantable stimulator can produce stimulus with >2 ms pulse width and stimulation with long pulse width might create tissue damage [9,10], (2) no GI implant is capable of sensing GI motility [11,12,13], and (3) bulky device size which increases surgical invasiveness.

**Figure 1:**
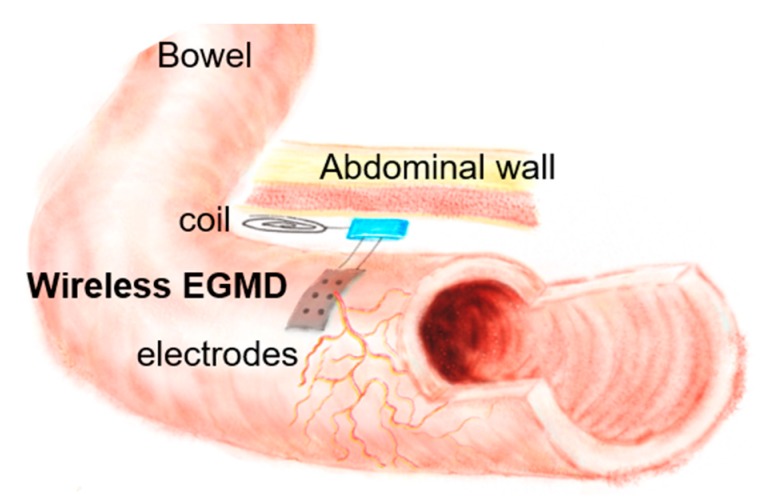
Conceptual illustration of wireless extraluminal gastrointestinal modulation (EGM).

In this work, we present a wireless extraluminal gastrointestinal modulation device (EGMD), aiming to address the aforementioned concerns to modulate and monitor GI motility (Figure 1). The implantable miniaturized, wireless EGMD incorporates a flexible electrode array to interface with the GI tract; a SoC as the core to perform stimulation, motility recording, and wireless power and data transfer through inductive links; as well as a heterogeneous packaging for device miniaturization. Both bench-top and in-vivo animal studies have been conducted to test and validate the EGMD.

The remainder of this paper is organized as follows: Section 2 briefly introduces GI physiology and electrophysiology. Section 3 addresses the design concerns for the wireless EGMD while the details and implementation of the EGMD will be presented in Section 4. The bench-top and in-vivo tests using rodent and porcine models and the experimental results will be described in Section 5. Section 6 draws the conclusion.

## 2. Gastrointestinal Physiology and Smooth Muscle Electrophysiology

**Figure 2:**
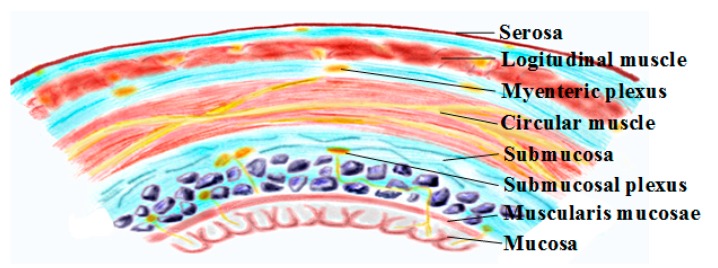
Cross-section of the gastrointestinal wall.

The gastrointestinal wall is organized as several concentric layers of specialized cells (Figure 2). From the lumen outward, the intestine can be divided into the mucosa, the submucosa, and the muscularis. Each intestinal layer performs a specialized function, but together they coordinate secretion, digestion, absorption, and peristalsis. In the intestinal muscularis, smooth muscle cells form two orthogonal layers of smooth muscle (circular muscle and longitudinal muscle) that are the effectors of intestinal peristalsis. Myenteric neurons are organized as two concentric networks, forming the submucosal and the myenteric plexuses while ICCs are embedded within each of the layers and interact with smooth muscle cells and the enteric nervous system. The contraction and relaxation of these smooth muscle cells are then controlled by the electrical activities of the myenteric neurons and ICCs: ICCs generate slow waves and enteric neurons fire spikes to induce GI smooth muscle contraction and relaxation. It is interesting to note that, although a slow wave is a spontaneous electrical rhythm with a varying frequency in different GI segments (e.g., 3 cpm in the stomach, 12 cpm in the duodenum, and ~8 cpm in the ileum), it alone does not always induce smooth muscle contraction [14]. Instead, contraction is generated when myenteric neurons are firing during the depolarization phase of the slow wave [15]. This thus makes electrical neuromodulation a viable treatment solution for gastrointestinal dysmotility to either induce smooth muscle contraction through direct muscle stimulation or the activation of myenteric network/ICCs indirectly via electrical stimuli.

## 3. System Design Consideration

The design consideration (e.g., wireless powering, recording, and stimulation) for the implantable devices targeting neuromodulation has been widely discussed in our prior works [16] and others [17,18,19]. However, the fundamental difference between nerve and GI tract stimulation inevitably introduces different design considerations and challenges for the GI implants, which include:

### 3.1. Versatile Stimulation Pattern

Due to the complex working mechanisms of GI motility, involving the enteric nervous system, smooth muscle cells, and ICCs, the effective stimulation protocols for inducing GI contraction is still inconclusive, and diverse stimulation parameters have been tried to engage different segments of the GI tract or even the same segment. For example, in the descending colon, it has been demonstrated that its contraction can be induced by 15 mA bipolar pulses with a pulse width of 3 ms [20] while stimuli with either 0.3 ms and 0.33 ms with different intensities have shown both capable of modulating stomach motility [21,22]. It has also been observed that stimuli with longer pulse widths are prone to recruit smooth muscle while those with short pulse widths excite the enteric neurons [23]. A prominent example is the Enterra GI stimulator that adopts 14 Hz, 300 μs, 5 mA pulse train to stimulate the stomach but it only demonstrates its efficacy in treating nausea, instead of affecting motility [24]. On the other hand, stimulation of the intestine wall using high amplitudes (i.e., 15~35 mA), and long pulse duration (e.g., 0.5~50 ms) results in thermal or electrolytic injuries of the tissue surrounding the electrodes [8]. Thus, in order to thoroughly investigate the optimal or effective safe stimulation/treatment protocols for modulating GI motility in different segments, an implant supporting a high diversity of stimulation parameters is desperately needed and is currently missing in the community.

### 3.2. Wireless GI Motility Recording

Real-time and quantitative measurement of GI motility would bring tremendous clinical benefit to assist clinicians in making medical decisions and also enables the realization of a closed-loop system that can adaptively adjust the stimulation pattern based on the patient’s varying GI response. In the current clinical setting, bowel sounds and passage of flatus/stool are considered as the benchmarks. However, recording bowel sounds requires frequent auscultation which is difficult in practice while the passage of flatus is also not ideal as some patients are not comfortable in reporting it and others may not be able to recall passing flatus. Furthermore, although several other techniques have been used in real-time GI motility measurement, incorporating them into a GI implant is less feasible due to the limitations of practicality, power consumption, and susceptibility to motion artifact. For instance, conventional force/pressure transducer and manometric recording used to assess motility are uncomfortable and might interfere with normal GI activity. A flexible polyimide-based piezoelectric device that transforms its mechanical deformation induced by gastric expansion/contraction into the voltage signal has been presented and validated in a porcine study, yet the recorded signal can be overwhelmed by motion artifact and its applicability for GI implant requires more thorough investigation [25]. The electrode–tissue impedance has also been shown effective to assess GI motility as the deformation of smooth muscle leads to the tissue impedance variation [26,27]. Nevertheless, sophisticated circuit blocks made by off-the-shelf components are used in [27] to estimate electrode–tissue impedance, inevitably increasing the power consumption as well as the footprint of the implant. In order to address the above mentioned issues, a hardware-efficient time-domain impedance analysis method is thus adapted from our prior work to perform quantitative measurements of GI motility [28,29]. The impedance measurement is derived by delivering high frequency current stimuli into the biological tissue and then measuring the electrode overpotential concurrently to acquire the electrode–tissue impedance variation. This high frequency stimuli enables the low-frequency motion artifact be separated from the signal of interest through low-pass filtering. This method has been first validated by recording the peristalsis wave through concurrent intraluminal pressure recording using a conventional sensor (SPR-751/940 Mikro-Tip catheter; Millar Instruments, Houston, TX, USA) and the proposed impedance measurement using rodent models [26].

### 3.3. Flexible, Electrochemically Safe Electrode and Miniaturized Package

Unlike vagus nerve or cortical implants that deal with nearly static biological tissue, the GI implant is facing a more demanding in-vivo environment as the smooth muscle moves constantly to propel the ingested food content. Therefore, the electrode array interfacing with the GI tract must be flexible to bear the contraction, relaxation, and peristalsis of the smooth muscle. A commonly adopted approach is stainless steel wire electrodes: the electrode is sutured subseroally for stimulation [30]. However, the number of suture sites increases when multiple electrodes are used, imposing challenges on the surgical procedures and possibly interfering with the normal activity of the GI segment. It is also critical to point out that existing GI implants are bulky (e.g., Enterra gastric stimulator has a volume ~37 cm^3^ [31]; EndoStim stimulator has a volume of 20.3 cm^3^ [32]; Arriagada et al. [27] presents a gastric stimulator with a volume of 53.4 cm^3^). It would thus be of clinical benefit to miniaturize the size of the implant as this not only reduces the surgical invasiveness but also possibly opens up new horizons of bioelectronic medicine to treat diseases associated with different organs or nerves by the miniaturized implant.

## 4. Wireless Extraluminal Gastrointestinal Modulation Device (EGMD)

**Figure 3:**
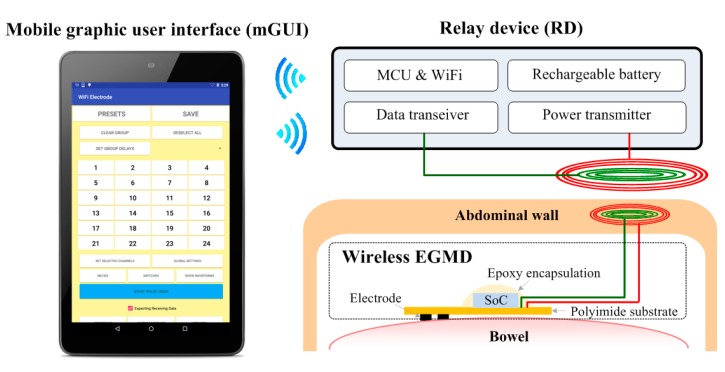
Wireless EGM system consists of a wireless extraluminal gastrointestinal modulation device (EGMD), a relay device (RD), and a mobile graphic user interface (mGUI).

Figure 3 shows the conceptual diagram of the wireless gastrointestinal motility modulation system, consisting of the proposed wireless EGMD, a tablet with a custom-made graphical user interface (GUI) to configure the EGMD, and a rendezvous device (RD) that links the EGMD and the users (i.e., scientists/clinicians/physiologists). In the application of treating GI dysmotility, a midline abdominal incision is performed to implant the EGMD: the EGMD and its electrode array are placed on the surface of the bowel to electrically stimulate and record the intestinal segment; a small subcutaneous pocket is created to house the coils for wireless power and data links; the RD is carried by the subject to deliver power and command to the EGMD; the users use the GUI to monitor the GI motility and set stimulation parameters accordingly. We are providing a system-level solution while the focus of this work would be placed on the wireless EGMD that integrates a unique wireless system-on-a-chip (SoC), a flexible electrode array, passive components, and coils into an unprecedentedly miniaturized device with versatile functionalities.

**Figure 4:**
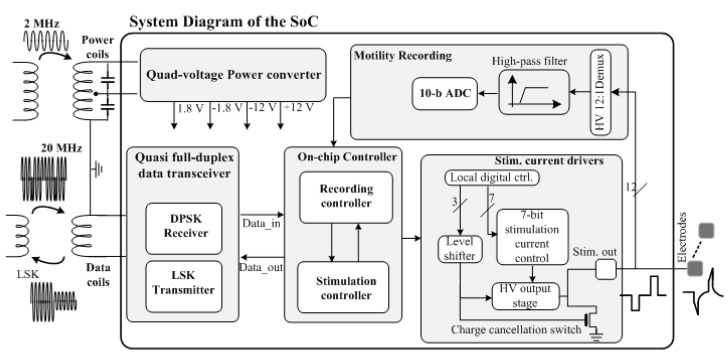
Functional block diagram of the system-on-a-chip (SoC) for wireless gastrointestinal (GI) stimulation and motility recording.

Figure 4 shows the functional block diagram and die photo of the wireless SoC capable of performing wireless powering, bidirectional data link, GI current-mode stimulation and motility monitoring, adapted from our prior work [33]. The on-chip power converter receives the inductive AC power signal to generate +/−1.8 V and +/−12 V, in which the low and high supply voltages are used to power the digital and wireless transceiver circuits, and the GI stimulator, respectively. The mixed-voltage design optimizes the system power consumption and performance as high compliance voltage for the stimulator is essential, allowing the user to investigate a wide range of parameters for GI stimulation. The current SoC supports stimulation intensities from 0.3 μA to 20 mA with a variable pulse width from 10 μs to a user-defined length, as well as a programmable stimulation frequency from 0.001 Hz to 200 Hz.

Furthermore, a differential-phase-shift-keying (DPSK) receiver and load-shift-keying (LSK) transmitter are adopted to implement the wireless transceiver [16]. The DPSK receiver takes the wireless command sent by the user. Subsequently, the on-chip controller (1) decodes the command and sets up the stimulator to generate the desired electrical stimuli, and (2) retrieves the recorded signal and encodes it for reverse data link through the LSK transmitter. It is worth noting that, instead of recording neural signals, bioimpedance measurement is performed through the SoC to monitor the GI motility. In the measurement, low-intensity current stimuli (e.g., several ~μA) with ~10–100 μs pulse width is applied to the GI segment and the resulting electrode overpotential is measured. A high-voltage (HV) multiplexer is implemented to allow the impedance recording among 12 channels (electrodes). Signal from the selected channel is then filtered through a high-pass filter to remove the low frequency motion artifacts and then digitized for further data encoding and wireless transmission as described in [33].

**Figure 5:**
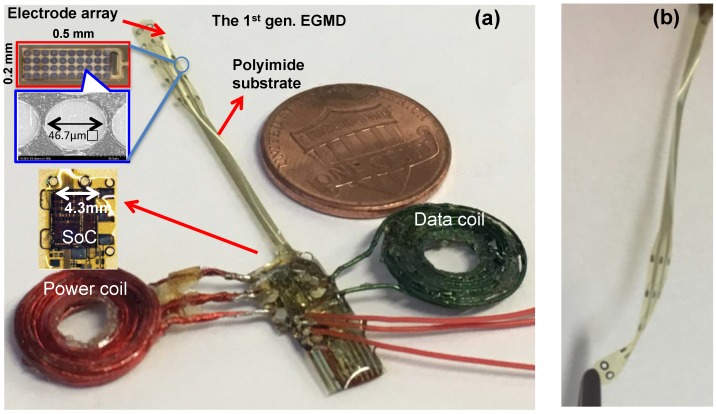
(**a**) Prototype of the miniaturized wireless EGMD. The high quality factor (Q) power coil is made by 100-strand litz wire (American Wire Gauge-48 (AWG-48)) with Q of >70 for high wireless power transfer efficiency and a data coil made of single wire (AWG-24) has low Q of ~5 to optimize data transmission bandwidth; (**b**) the flexible electrode array.

By adopting the heterogeneous packaging and electrode fabrication technique developed by us [34], the wireless EGMD with a multi-electrode array is miniaturized into an unprecedented small form factor, compared to all other GI implants (Figure 5). Each electrode has a geometric size of 0.5 mm × 0.2 mm and surface roughness process is conducted to increase the effective surface area in order to increase its charge storage capacity (CSC). The wireless EGMD then integrates the SoC, electrode array, and coils on an 8-μm-thick polyimide substrate. The bond pads, electrodes, and interconnects on the flexible substrate are fabricated using e-Beam evaporation deposition as platinum and titanium layers with thickness of 10 nm and 200 nm, respectively. All necessary electrical connection to enable the function of the SoC is made through the patterned metal trace on the polyimide substrate, serving as a flexible interposer. The SoC is then connected to the polyimide substrate via gold bumps. Coils and other external components (e.g., capacitors) are connected to the contact of the substrate pads using silver-filled conductive epoxy (EPO-TEK H20E, Epoxy Technology Inc., Billerica, MA, USA). The electrode array has a length of 4.3 cm and a width of 8 mm. Suturing holes are fabricated at the tip of the array for the surgeon to suture the electrode onto the bowel. After the integration, the device is encapsulated with biocompatible insulating epoxy (3M DP100 Plus Clear, 3M Corp., Maplewood, MN, USA). This encapsulated wireless EGMD has a miniaturized size with a weight of ~0.7 g and a volume of ~0.5 cm^3^, when coils and the SoC are placed coaxially.

## 5. Experimental Results

Three experiments are conducted: a bench-top test to validate the functionality of the assembled device, and two in vivo experiments using rodent models and porcine models.

**Figure 6:**
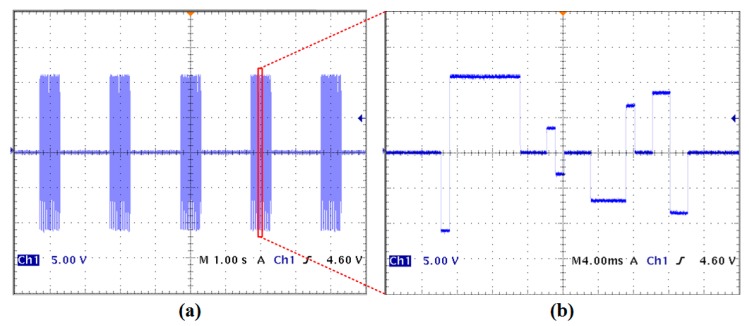
Measured stimulation waveform that demonstrates the high programmability of the stimulator. (**a**) Pulse train stimulation with 0.6 s ON time and 1.4 s OFF time. (**b**) Zoom-in view of (**a**).

The setup of the bench-top test is derived from our previously developed system for spinal cord implants [33]. Wireless power and data communication were built through our customized Class-E power and data transceiver made with off-the-shelf components to configure the EGMD. The user-defined command is issued through an in-house developed GUI based on C# (Microsoft Inc., Seattle, WA, USA). The output waveform of the stimulator (Figure 6) demonstrates its capability of generating versatile patterns for GI tract stimulation. A current pulse train containing various current intensities, pulse widths, and different leading polarities with 0.6 s ON time and 1.4 s OFF time is applied to a 550 Ω resistor (Figure 6a). The stimulation ON and OFF time can also be arbitrarily set by the user, depending on the application of interest. Figure 6b shows the zoom-in view of the pulse train, which exhibits an example of combining four stimuli with intensities ranging from 6 mA to 20 mA and pulse widths ranging from 1 ms to 8 ms. It should also be noted that the charge imbalance shown in Figure 6b will not occur in reality when performing GI tract stimulation as the purpose of the sample waveform shown is to demonstrate the high programmability of the stimulator. This high flexibility would facilitate the researchers in investigating the currently inconclusive therapeutic efficacy of electrical stimulation on GI dysmotility.

**Figure 7:**
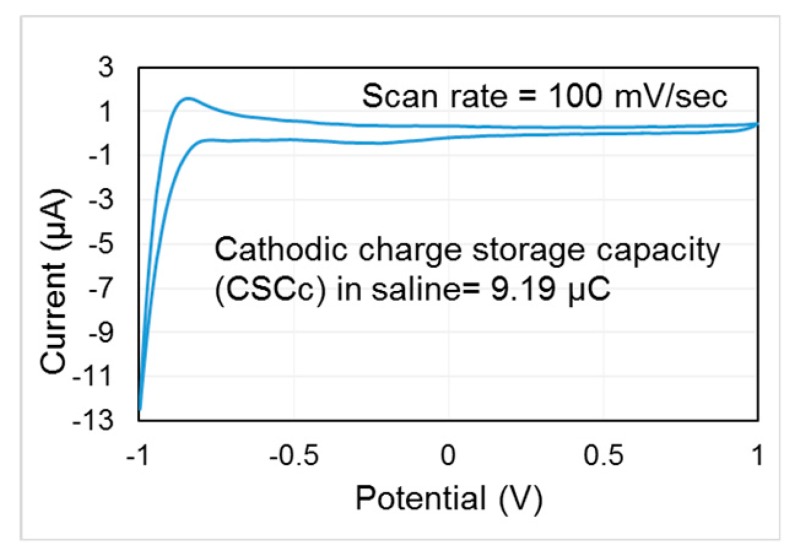
Measured cyclic voltammogram of the electrode array.

The cyclic voltammetry (CV) test on the fabricated electrode array is conducted to investigate its safe charge delivery limit in order to avoid any undesired electrochemical reaction that might damage tissue or electrodes during stimulation. The PalmSens3 system (PalmSens, GA Houten, The Netherlands) is used for the test under standard three electrode set-up: working electrode (the fabricated electrode), AgCl electrode (reference electrode), and platinum electrode (counter electrode). Electrodes are immersed into the 0.1 M NaCl solution to mimic the in-vivo environment. A linear ramp signal at a scan rate of 100 mV/s is delivered to the electrode. The measured cyclic voltammogram is shown in Figure 7. The water window of the electrode is [−0.9 V 1 V]. The charge storage capacity (CSC) of this electrode is calculated as 9.19 µC by integrating the closure area of the cyclic voltammogram. The estimated CSC thus sets the constraints to ensure the delivered charge under any combination of stimulation parameters (i.e., stimulation current intensity and pulse width) should not exceed 9.19 µC, providing a guideline for our animal study using the electrode.

**Figure 8:**
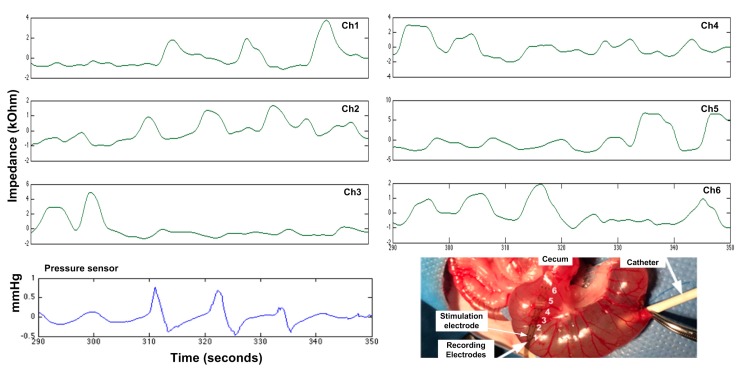
Simultaneous motility and pressure recording from a normal segment. The bottom-right inlet shows the in vivo experiment setup. Six electrodes on the right column with a separation of 3.3 mm were used for motility recording. Only the change is impedance is of interest to indicate motility.

In vivo experiments were first conducted using adult female Lewis rats under isoflurane anesthesia. All procedures comply with the Use of Laboratory Animals and were approved by the Animal Research Committee at University of California, Los Angeles (UCLA). In this pilot experiment, we first tested the feasibility of using the SoC and the flexible electrode array to investigate GI motility. Laparotomy was performed to expose the cecum of a healthy rat. The electrode array were placed on top of the cecum and connected to the SoC. A tube was then inserted for saline injection and to house the pressure sensor, whose tip was beneath electrode #2 (Figure 8). Stimulus of 1 mA, and 1 ms at 100 Hz was delivered through the stimulation electrode to induce peristalsis in the cecum while multiple stimuli with the parameter of 3 μA, and 0.1 ms at 100 Hz were sent to each of the recording electrodes for motility recording. Motility was recorded from multiple electrodes and it can be seen that the recorded pressure is directly correlated to the motility change on electrode #2, validating the applicability of the proposed method.

**Figure 9:**
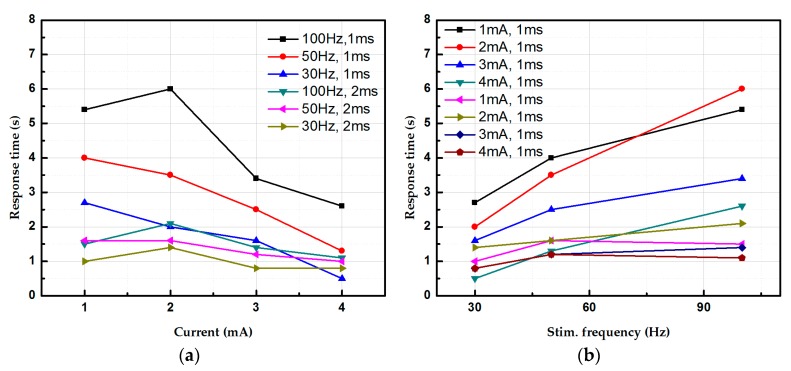
Response time of smooth muscle contraction under different stimulation parameters in rodent models. Response time is defined as the time difference between the end of stimulation and onset of smooth muscle contraction (**a**) Response time under different current intensities. (**b**) Response time under different stimulation frequencies. The electrical charge delivered into the bowl is within the safe limit of the electrode.

Subsequently, the rat in the experimental group underwent jejunal segment isolation with benzalkonium chloride (BAC) aganglionosis induction and the rat in the control group underwent a sham laparotomy. The surgical procedure for the rat study can also be found in [35]. Niflumic acid was further applied to block slow waves in both normal and aganglionic bowels. Constant frequency stimulation (i.e., 30–100 Hz) with different pulse widths and stimulation intensity were delivered to the intestine (i.e., normal, aganglionic intestinal, and slow-wave-blocked intestinal segments were used) while electrodes on the array captured local contraction and propagation of intestinal motility. No contraction was observed before stimulation in the aganglionic bowel. Impedance and stimulation-to-response time (time difference between stimulation and muscle contraction onset) were both measured (Figure 9). In this study, local contraction underneath the stimulating electrode and a peristaltic wave of contraction were only observed in normal intestines and colons; only local contraction was found in aganglionic and slow-wave blocked bowels, suggesting that enteric neurons or ICCs were absent to induce peristalsis. Response time (RT) dropped when stimulation intensity was increased from 1 mA to 4 mA, but increased when stimulation frequency varied from 30 Hz to 90 Hz in normal bowel stimulation. This suggests different neural circuitries were activated per different stimulation parameters. Similar trends were observed in aganglionic bowels, but there were no significant changes in RT of slow-wave-blocked normal and aganglionic bowels.

**Figure 10:**
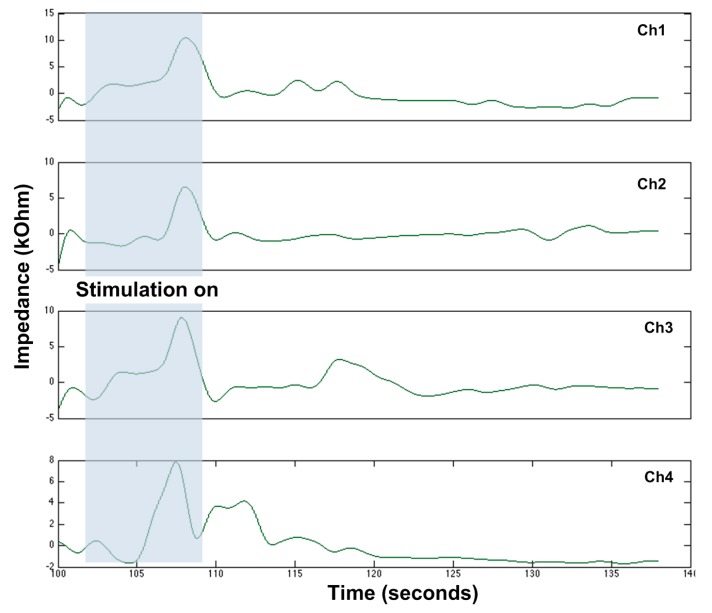
GI motility recording from the porcine model through impedance variation. Stimulus was applied to electrode #4, from *t* = 102 to *t* = 109. The measured results during this window are artifacts. Ch4 presents a contraction after *t* = 109.

Subsequently, a pilot study using porcine models was conducted. The use of all animals was approved by the Animal Research Committee (institutional review board No. 2014-142-03E). Juvenile mini-Yucatan pigs weighing 8–12 kg were premedicated with 15 mg/kg ketamine, 0.5 mg/kg midazolam, and 0.3 mg/kg meloxicam administered intra-muscularly. Pigs were then intubated and maintained under general anesthesia with 1–3% inhaled isoflurane. Maintenance fluids were administered at 10 mL/kg/h. A midline laparotomy was performed and a short segment of jejunum was identified and externalized. The electrode of the EGMD was then placed onto the serosal surface of the intestine for stimulation and recording. In this pilot study, two stimuli with the parameters of (1) 100 Hz, 2 ms, and 4 mA and (2) 100 Hz, 0.1 ms, and 3 μA were delivered to the pig’s jejunum to induce smooth muscle contraction and motility measurement. The recorded motility from four electrodes in a row of the EGMD is shown in Figure 10, which shows the impedance variation during the onset of smooth muscle contraction/relaxation. Only local contraction with no peristalsis was observed, reflected from both the electrode–tissue impedance variation and our visual inspection. A thorough study using the porcine model is still ongoing to investigate the optimal stimulation parameters and the efficacy of the EGMD in improving GI motility after implantation in surviving animals.

## 6. Conclusions

A summary and comparison with other GI implants/devices is shown in Table 1. The proposed wireless EGMD integrates the functionalities of wireless power transfer and data communication, versatile stimulation, and a unique method of motility recording into a miniaturized device through our heterogeneous system-in-a-package. The developed flexible electrode/SoC/EGMD have also been validated and tested both in bench-top and in animal studies. The versatile stimulator not only allows the researcher to investigate effective stimulation parameters on various GI segments for deciphering the complex autonomic/enteric nervous system, but also has a therapeutic potential once the effective parameters are identified. The proposed real-time motility monitoring further conveys the causality between GI stimulation and smooth muscle activities that can significantly help the clinicians/researchers optimize the stimulation protocols, toward personalized neuromodulation. Moreover, unlike in other works that adopt long stimulation pulse and high stimulation intensity—which raise concerns in thermal and electrolytic injuries around the stimulation site and the power consumption of the implant [8]—we have shown that stimuli with short stimulation pulse and stimulation current intensity of several mAs is capable of inducing bowel contraction, either through directly activating the smooth muscle or the enteric neuron network. The EGMD also has the potential to be further expanded to other neuromodulation applications, such as neurmodulation on the stomach, bladder, heart, vagus nerve, and other internal organs as bioelectronic medicines, and can be further served as research tools for various biomedical applications to investigate underlying biological mechanisms and the study of novel electroceuticals for different diseases.

## Figures and Tables

**Table 1:** Comparison table of the GI stimulator.

**Reference**	[29]	[30]	[25]	[36]	[37]	This Work
**Power source**	Battery ^1^	Battery ^1^	Battery ^1^	Battery ^1^	Wireless power	Wireless power
**Stimulation mode**	Current	Current	Voltage	Current	Current	Current
**Amplitude (mA)**	5	2–10	4–10	5	5	0.003–20 ^2^
**Stimulation frequency (Hz)**	2.1–130	2–80	10–10	2.1–130	14	User defined
**Stimulation pulse width (ms)**	0.06–0.45	0.03–0.975	1–100	0.06–0.45	0.33	0.01–User defined
**Compliance voltage (V)**	10.5	N/A	10	3	3.3	±12
**Continuous stimulation**	Yes	No	No	N/A	No	Yes
**Pulse train stimulation**	Yes	Yes	Yes	Yes	Yes	Yes
**Residual charge removal**	N/A	N/A	N/A	N/A	N/A	Yes
**Motility recording**	No	No	Impedance	No	EGG	Impedance
**Implant size (cm^3^)/weight (g)**	37/42	20.3/28.5	53.4/48.9	3.3/3.1	2.8/5.2	~0.5/~0.7

^1^ Nonrechargable battery. ^2^ Multiple stimulator outputs can be combined to support large stimulation current.
